# Balloon expandable transcatheter aortic valve implantation with or without pre-dilation of the aortic valve – rationale and design of a multicenter registry (EASE-IT)

**DOI:** 10.1186/1471-2261-14-160

**Published:** 2014-11-18

**Authors:** Peter Bramlage, Justus Strauch, Holger Schröfel

**Affiliations:** Institute for Pharmacology and Preventive Medicine, Bahnhofstrasse 20, 49661 Cloppenburg, Germany; Clinic for Cardiosurgery and Thoracic Surgery, Berufsgenossenschaftliches Universitätsklinikum Bergmannsheil, Bochum, Germany; Clinic for Cardiac Surgery, Karlsruhe, Germany

**Keywords:** EASE-IT, Transcatheter aortic valve implantation, TAVI, Balloon aortic valvuloplasty, BAV

## Abstract

**Background:**

In patients with severe calcific aortic stenosis, balloon aortic valvuloplasty (BAV) is routinely performed in order to pre-dilate the stenosed aortic valve prior to transcatheter aortic valve implantation (TAVI). Although pre-dilation is considered to be essential for the preparation of the valve landing zone, there is no clear evidence to support its clinical value. In contrast, BAV has been suggested to be linked to several complications. Notably, while preliminary evidence has supported the feasibility and safety of TAVI without pre-dilation, larger studies directly comparing the benefit/risk profile of TAVI in the presence and absence of pre-dilation are required.

**Methods/Design:**

Therefore, a prospective, two-armed, multicenter registry (EASE-IT) was designed to obtain essential data concerning procedural success rates, adverse events, and mortality in a large cohort of patients undergoing transapical (TA)-TAVI using the Edwards SAPIEN 3 balloon expandable heart valves with and without pre-ballooning.

**Discussion:**

Data provided by EASE-IT will be used to assess the relevance of BAV during the TAVI procedure and to investigate associations between patient characteristics and outcomes. Therefore, results obtained from the EASE-IT registry could contribute to reduced rates of TAVI-associated morbidity and mortality in patients with severe, calcific aortic stenosis.

**Trial registration:**

ClinicalTrials.gov Identifier: NCT02127580

## Background

Degenerative aortic stenosis (AS) is characterized by the narrowing of the aortic heart valve and most frequently is associated with progressive calcification and fibrosis of the valve leaflets. It represents the most common form of valve disease in the Western world and is associated with an estimated 5-year survival rate of 32% in the absence of treatment [1–3]. Thus, severe symptomatic AS constitutes a class I indication for aortic valve replacement surgery [4]. In this regard, conventional surgery has proven to be safe and effective for eligible patients [5, 6]. However, open heart surgery cannot be performed in 30–40% of patients due to advanced age and/or comorbidities [1, 7]. For this reason, transcatheter aortic valve implantation (TAVI), which was first described by Cribier et al. [8, 9], has emerged as a ground-breaking alternative technique for valve delivery in elderly, mostly fragile patients. Indeed, the TAVI approach has shown similar efficacy as compared to surgery and survival benefits in patients where surgery cannot be performed (PARTNER) [10–12]. Since its introduction into clinical use, continuous efforts have been undertaken to evaluate and improve TAVI techniques with the objective of reducing TAVI-associated complications.

Since the introduction of TAVI [8, 9], pre-dilation of the aortic valve has been considered as an obligatory step prior to transcatheter heart valve (THV) placement. BAV is carried out under rapid right ventricular pacing (>180 bpm) for up to 30 seconds in order to facilitate the crossing of the aortic annulus and to enable full balloon-inflation for 3–5 seconds with the associated obstruction of the aortic annulus. Potential advantages of performing BAV before replacement of the aortic valve may be the following: sizing evaluation when injecting above the inflated balloon, checking the risk for coronary occlusion and supporting evidence for coronary protection in selected cases, checking for pacemaker capture in real-time, checking for “balloon jump” in cases with LVOT obstruction or mitral prosthesis, and checking the synchronization of operating team [13]. Though, BAV has been suggested to be associated with the following serious complications [14–17]: (1) hemodynamic failure such as prolonged hypotension, need for cardiopulmonary reanimation, cardiac tamponade; (2) arrhythmia requiring medical treatment or AV block with need for pacing; (3) vascular events like systemic embolism or myocardial infarction; and (4) bleeding due to cardiac perforation, trauma-mediated aortic root rupture etc. Therefore, due to these potentially life-threatening complications, it has been suggested that removal of the pre-dilation step might reduce the rates of adverse events in patients undergoing TAVI [18–20].

So far, only preliminary studies have investigated the clinical value of pre-dilation of stenosed aortic valves in patients receiving self-expandable THVs through the transfemoral (TF) route [18, 19] or balloon expandable valves via the transapical (TA) route [20]. In a pilot study by Grube et al., the feasibility and safety of TF-TAVI in the absence of pre-dilation was studied using the self-expandable Medtronic CoreValve (MCV) device in 60 consecutive patients within 13 centers [18]. A technical success rate of 96.7% was observed, with post-dilation required in 16.7% of the cases and an in-hospital mortality rate of 6.7%. Thus, the procedure was reported to display a safety and efficacy profile that was similar to that of the standard approach with dilation. In addition, Mendiz et al. conducted a study of 51 patients undergoing TF-TAVI without pre-dilation using the MCV device within a single center, observing a procedural success rate of 94.2% and an in-hospital mortality rate of 3.9% [19]. However, only one pilot study involving six patients has examined TA delivery of balloon expandable Edwards SAPIEN THVs in the absence of BAV [20]. Successful deployment was observed in all patients and elimination of the pre-dilation step was suggested to result in shortened procedural duration, decreased radiation exposure, and reduced usage of contrast agents. It was also proposed that TA-TAVI without BAV might diminish the likelihood of hemodynamic instability in patients with impaired LVEF.

Although these preliminary investigations have started to evaluate the safety of TAVI without pre-dilation, larger datasets directly comparing TAVI in the presence and absence of BAV are required to fully assess the risks and/or benefits associated with elimination of the pre-dilation step. For this reason, we have designed a prospective, two-armed, multicenter registry (EASE-IT) to compare the safety and efficacy of TA-TAVI using Edwards SAPIEN 3 with and without pre-ballooning. EASE-IT aims to document adverse events, mortality, and procedural data in order to assess the importance of pre-dilation during TAVI and to identify potential associations between patient characteristics and outcomes.

### Methods/Design of EASE-IT

EASE-IT is a multicenter, prospective, two-armed, observational registry. Enrollment of 200 subjects will take place at up to 10 study-sites in Germany. Approval was obtained by the Ethics Committee of the Landesärztekammer Stuttgart on February 25^th^ 2014 and confirmation obtained at each site participating in ROUTE prior to patient enrollment. All patients are required to provide written informed consent prior to participation. The investigation commenced in April 2014.

### Site selection

Sites are selected based on prior experience with TA-TAVI (i.e., minimum of 20 prior implantations) and the ability to enroll a minimum of two patients per month. All participating sites have been, independent of this registry, trained in the use of the Edwards SAPIEN 3 and Ascendra Balloon Catheter according to the manufacturer’s instructions for use (Edwards Lifesciences, Irvine, CA, USA) and by means of exhaustive fundamentals training (i.e., didactic sessions, case observations, device preparation, and simulator training). This will be followed by on-site training as specified by the Edwards Standard Operating Procedure.

### Patient selection

Patient eligibility for EASE-IT will be based on the following criteria: (1) indication for TAVI in accordance with the Edwards SAPIEN 3 instructions; (2) eligibility for TAVI with and without BAV; and (3) at least 18 years of age. Patients with contraindications based on instructions for use of either TAVI or the Ascendra Balloon Catheter will be excluded from the study. Additionally, patients meeting any of the following criteria will be excluded: (1) logistic EuroSCORE I >50%; (2) mitral or tricuspid valvular insufficiency (> grade II); (3) previous aortic valve replacement; (4) uncontrolled atrial fibrillation; (5) left ventricular or atrial thrombus by echocardiography; (6) recent cerebrovascular event (within the last 3 months);and (7) high probability of non-adherence to follow-up requirements (due to social, psychological, or medical reasons). Each participating center must document 10 consecutive cases with BAV and 10 cases without BAV.

### Procedure

Notably, TA-TAVI with pre-dilation is to be performed as previously described by Walther et al. [13], whereas the procedure without pre-dilation will be carried out in a similar manner to that described by Wendler et al. [20]. Pre-operation computed tomography will generally be used for valve sizing purposes. However, investigators can modify these procedures to fit their needs. Patients will be assigned into groups (i.e., with BAV or without BAV) by their physician prior to study enrollment and independent of the registry.

### Pre-defined objectives

**Figure 1:**
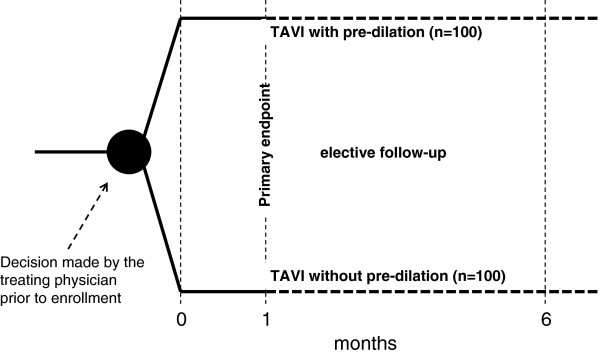
**Registry design.**

The primary evaluation criteria at 1 month will consider the combined endpoint of all-cause mortality, stroke, non-fatal myocardial infarction, acute kidney injury, and pacemaker implantation within 30 days after TAVI as to VARC2 (Figure 1). Secondarily, the rates of each of these individual adverse events will be considered, along with duration of radiation, amount of contrast agent, and aortic root rupture. Six months after TAVI, the combined and separate adverse events will once again be evaluated. Patient outcomes will be compared between the two groups, with implantation plus pre-dilation considered as the control arm.

### Data collection

Patient data will be collected using electronic case report forms (eCRFs), which must be signed electronically by investigators and/or designees (Table 1). The eCRFs should be completed at the earliest opportunity. All registry documents will identify subjects by patient registry identification numbers only (or initials when applicable).

**Table 1:** **Data collection**

Parameter	Admission	Intervention	Discharge	30d FU	6 Mo FU
Inclusion/Exclusion criteria	X				
Demographics	X				
Diagnosis of valve disease	X				
Echocardiography	X				
Symptoms	X			X	X
Cardiac baseline characteristics	X				
ECG	X		X		
Comorbidities	X				
Risk scores	X				
Prior cardiovascular intervention	X				
Current medications	X		X	X	X
Interventional details		X			
Interventional results		X			
AE		X	X	X	X
Hospitalization duration			X		
Creatinine value	X		X		
Early safety/Clinical efficacy				X	X

A total of 20% of the sites (i.e., up to two sites) will be selected at random for monitoring following completion of patient follow-up and documentation. Source data verification will be performed for all patients in these centers. Also, 100% source data verification will be conducted for the following serious adverse events: death, stroke, major bleeding, valve complications, vascular complications, permanent pacemaker implantation, and renal failure. For data analysis, linearized rates and actuarial probability statistics will be used for adverse event reporting, and Kaplan-Meier analyses will be performed for survival and adverse event outcomes when appropriate.

### Statistics

The sample size for EASE-IT was established based on previously reported event rates and the feasibility to detect an absolute risk reduction for the primary endpoint of 13% at 30 days while taking into consideration a 10% drop out rate [18, 20]. Intention-to-treat analysis, based on all patients enrolled in the registry will be performed. Subjects are considered registry participants when they enter the cath lab/hybrid suite. Descriptive data summaries will be used to present and summarize the collected evaluation data. For categorical variables (e.g. gender) frequency distributions will be given. For numeric variables (e.g. patient age) minimum, maximum, mean, median and standard deviation will be calculated. Linearized rates and actuarial probability statistics may be used where appropriate for adverse event reporting. Kaplan-Meier analysis will be performed for survival outcomes and where appropriate for adverse event outcomes.

## Discussion

EASE-IT represents a prospective, multicenter, two-armed registry aimed at documenting the course of patients undergoing TA-TAVI using the Edwards SAPIEN 3 with or without pre-dilation via BAV. Information provided by EASE-IT will be used to assess the value of pre-dilation during TAVI procedures and to investigate associations between patient characteristics and outcomes. Thus, results obtained from the EASE-IT registry should have several important implications.

Although recent pilot studies have preliminarily suggested the feasibility and safety of performing TF-and TA-TAVI without pre-dilation of the stenosed aortic valve [18–20], these studies have not directly compared the outcome of patients undergoing TAVI with and without pre-dilation. Nevertheless, in the study by Grube et al. [18], 60 subjects undergoing TF-TAVI using the MCV device in the absence of BAV were compared to a historical control group (with BAV), suggesting that the efficacy of the simplified procedure (i.e., without pre-dilation) was similar to that of the standard approach. However, it is known that this type of comparison can be associated with several methodological limitations. Therefore, the conclusions by Grube et al. must be appropriately verified. In addition, data regarding delivery of the Edwards SAPIEN THV in the absence of pre-dilation are extremely limited [20]. For these reasons, EASE-IT has been designed to determine whether BAV during TA-TAVI using the Edwards SAPIEN 3 yields relevant beneficial or negative procedural effects based on a two-armed registry approach, which will allow for direct comparison between the patient groups (i.e., with BAV *vs.* without BAV). Thus, the comparative analysis that can be performed from information collected during the EASE-IT registry will be fundamental for establishing the importance of pre-dilation during TA-TAVI.

Notably, a recent imaging study suggested that new silent cerebral lesions appear in approximately 90% of patients undergoing TAVI [21], with the highest number of embolisms shown to occur at the time of pre-dilation and valve deployment [22]. This is interesting considering that BAV has been suggested to cause thrombosis, which can result in coronary obstruction, myocardial infarction, or stroke [16]. In addition, BAV has been linked to transient ischemia (coronary, cerebral, and renal), aortic root rupture, as well as hemodynamic failure and/or systemic inflammation in patients with reduced LVEF [14–16, 23]. With regard to these BAV-related complications, EASE-IT should yield important information concerning the benefit of pre-dilation during TAVI. Indeed, if the results of EASE-IT demonstrate that BAV during TA-TAVI is unnecessary or detrimental, then these risks could be avoided altogether by elimination of the pre-dilation step. On the other hand, if BAV is found to contribute to procedural success, then exposure to these potential BAV-associated risk factors may be warranted. Also, it is possible that BAV will yield differential benefits or procedural risks within distinct populations (e.g., detrimental for patients with reduced LVEF). For this reason, EASE-IT will also examine associations between patient characteristics and outcomes. Thus, results obtained from the EASE-IT registry have the potential to promote evolution of the TAVI procedure in order to reduce the risk of microembolization, stroke, and other severe complications in patients with AS.

Regarding exclusion of the pre-dilation step during TAVI, the resulting simplified approach might reduce the procedural time, amount of contrast agent used, and duration of fluoroscopy as indicated by Wendler et al. [20]. In this regard, as part of the secondary evaluation criteria in EASE-IT, information related to these procedural factors will be recorded for analysis. Indeed, the simplified approach would also eliminate the delay between pre-dilation and valve deployment, which represents a time during which patients can become hemodynamically unstable. Thus, removal of the pre-dilation step has been suggested to be ideal for avoiding complications in patients with severely impaired LVEF [20]. Nevertheless, additional risks may be associated with implantation of the valve in the absence of pre-dilation, such as the potential need for post-procedural dilation, which was found to be required in 16.7% of subjects in the absence of BAV by Grube et al. [18]. Thus, EASE-IT will be important for analyzing the potential benefits or disadvantages associated with altering procedural factors during the simplified TAVI approach.

Furthermore, results obtained by EASE-IT regarding the importance of pre-dilation during TAVI might only be applicable to the Edwards SAPIEN 3, and not other commercially available devices. In this regard, another ongoing prospective, randomized trial has been initiated by a group at Bonn University (NCT01539746; Transcatheter Aortic Valve Implantation Without Pre-dilation [SIMPLIFy TAVI]) in order to investigate the 30-day composite endpoint in patients with severely impaired LVEF (≤35%) undergoing TAVI using MCV devices. Therefore, EASE-IT registry results, which are likely to be reported after completion of the SIMPLIFy TAVI trial (scheduled to end December 2014), should hold particular significance with regard to the generalizability of the impact of BAV on TAVI outcome as related to distinct devices and populations. Indeed, studies testing the importance of the pre-dilation step will need to be carried out for specific delivery systems, routes, and patient populations in order to conclusively determine whether the use of BAV is beneficial. Therefore, EASE-IT will be essential for contributing to the body of data necessary for establishing the value or risk associated with pre-dilation procedures during TAVI.

### Potential limitations of EASE-IT

Although EASE-IT represents a critical step for examining the importance of performing pre-dilation during TAVI procedures involving Edwards SAPIEN 3, the study might display limitations related to its design. Indeed, the non-randomized nature of the registry could lead to potential bias, as investigators will assign patients into the “with BAV” and “without BAV” groups at their discretion while patients are eligible for either route. In addition, because we ask for patients eligible in principal for TAVI with AND without BAV we will miss those only eligible for one of the routes. This cannot be avoided, however, if we want to compare both techniques in one patient population. We have chosen to use the observational design because we only incompletely know the variables affecting the decision and are thus not able, without a pilot documentation like the present one, to properly assess the variables necessary for the decision and these might deserve a prospective assessment in an upcoming randomized controlled trials.

Nevertheless, inclusion into the EASE-IT trial requires that patients be eligible for TAVI with and without BAV, which could alleviate the potential for bias. In addition, registry data tend to be less complete when compared to randomized clinical trials. However, in this regard, 20% of the sites will be randomly selected for monitoring following completion of patient follow-up and documentation. Source data verification will be performed for all patients in these centers. Moreover, it is possible that the 6-month follow-up will not be sufficient to detect differences in long-term adverse outcomes between the groups with and without pre-dilation. Thus, future randomized clinical trials may be needed in order to assess the long-term effects associated with eliminating BAV from the TA-TAVI procedure.

## Conclusions

The EASE-IT registry will provide essential data concerning procedural success rates, adverse effects, and mortality in a large cohort of patients undergoing TA-TAVI with and without pre-dilation. This information is essential for determining the benefits or complications that might be associated with the pre-dilation step during TAVI. Therefore, results obtained from EASE-IT could contribute to reducing rates of TAVI-associated morbidity and mortality in patients with severe AS.
